# Fertile field emission response in scrambled SmNiO_3_ nanopins

**DOI:** 10.1038/s41598-026-37508-9

**Published:** 2026-04-11

**Authors:** Subrata Karmakar, G. Anil Kumar, K. Kumar Angajala, Chetan D. Mistari, M. Amrutha, Pallab Kumar Sarkar, Saif Taqy, Brahmananda Chakraborty, M. A. More, Ariful Haque

**Affiliations:** 1 Department of Physics, Manipal University Jaipur, Jaipur, 303007 Rajasthan India; 2 Electrical Engineering, Ingram School of Engineering, Texas State University, San Marcos, TX 78666 USA; 3 Department of Physics, Sreenidhi Institute of Science and Technology, JNTU, Hyderabad, 501 301 India; 4 Department of Chemistry, Vardhaman College of Engineering, Shamshabad, Hyderabad, Telangana 501218 India; 5 R. H. Sapat College of Engineering, Nashik, 422005 Maharashtra India; 6 Department of Physics, Karpagam Academy of Higher Education (Deemed to be University), Coimbatore, 641021 India; 7 Homi J Bhabha National Institute, Bhabha Atomic Research Centre, Trombay, 400085 Mumbai India; 8 High Pressure & Synchrotron Radiation Physics Division, Bhabha Atomic Research Centre, Trombay, 400085 Mumbai India; 9 Department of Physics, Savitribai Phule Pune University, Pune, 411 007 India; 10 Materials Science, Engineering & Commercialization Program, Texas State University, San Marcos, TX 78666 USA

**Keywords:** Strongly correlated perovskite, Nanopins, Electric field emission study, Stability, Density functional theory, Materials science, Nanoscience and technology, Physics

## Abstract

This present work focuses on the synthesis of scrambled SmNiO_3_ nanopins by the hydrothermal route, structural and morphological characterizations, and observing their potential electric field emission response with density functional theory (DFT) study. The Rietveld refinement of X-ray diffraction (XRD) and Raman characterization study reveals an orthorhombic phase (space group *Pnma*, No. 62) of SmNiO_3_, along with Raman-active NiO_6_ octahedral vibrational modes and Sm-O lattice motion. The high-resolution scanning and tunneling electron microscopy images exhibit monodispersed hierarchically self-assembled pin-like surface morphology with a crystal lattice spacing of 3.16 Å for 110 planes, which offers exceptional field emission response. The chemical states of Sm, Ni, and O were interpreted by X-ray photoelectron (XPS) spectroscopy in +3 oxidation states (4f^5^ configuration), mixed Ni^3^⁺/Ni^2^⁺ valence states, and -2 lattice and defect states, respectively. The current density (J) vs. electric field (E) plots demonstrate a maximum field emission current density ~ 30 µA/cm^2^ @ 8.4 V/µm, and very enduring field emission current stability was observed over a long period of time, 3 h 20 min, with bright field emission fringes. Very low turn-on (E_turn-on_) and threshold (E_threshold_) were discerned ~ 5.1 V/µm @1 µA/cm^2^ and ~ 7.64 V/µm @10 µA/cm^2^ from J-E curves. The local work function (φ) was calculated to be ~5.13 eV by density functional theory (DFT) calculations, and the field enhancement factor (β) was measured to be ~3343. The noteworthy electric field emission response, low turn-on field, and long-term stable field emission enable SmNiO_3_ nanopins to be a high-efficiency electron source for field-emitting display (FED), X-ray source, vacuum transistor device, etc.

**Supplementary Information:**

The online version contains supplementary material available at 10.1038/s41598-026-37508-9.

## Introduction

Perovskite materials are becoming more and more significant than conventional ceramic or composite materials due to their enhanced physical and transport characteristics, and they generate a lot of interest in engineering, physics, chemistry, and science^1,2^. The advanced distinctive benefits of the ABO_3_ type perovskite oxides, in particular, such as their oxygen vacancy and oxygen octahedral structure, ferromagnetic effects, improved electrical conductivity, increased corrosion resistance, and exceptional chemical stability, have sparked significant study interest in recent scenarios^3,4^. Among various perovskite ABO_3_ structure materials, the LnNiO_3_ (where Ln = La, Pr, Nd, Sm, and Lu), have been reported in a variety of applications, such as solid-electrolyte batteries, solar batteries, supercapacitor, and other electrochemical energy storage devices, due to their alluring advantages of being affordable, abundant, low in pollution, easy to synthesize, and having outstanding electrochemical performance^5–10^. The rare-earth SmNiO₃ (SNO) is a strongly correlated perovskite nickelate (RNiO₃ family) with a high coupling between the lattice, charge, and orbital degrees of freedom, a temperature-driven metal–insulator transition (MIT), and a considerable tunability by strain, stoichiometry, and doping^11–13^. The SmNiO_3_ is a perovskite-type distorted rare-earth nickelate with an orthorhombic crystal structure and transformed to monoclinic (P2₁/n) below the metal-to-insulator (MIT) transition. Recently, the SmNiO_3_/BaTiO_3_ superlattices exhibits a interplay of metallicity, ferroelectricity and Nd doping in SmNiO_3_ makes a smart thermal-adaptive perovskite for next-generation aerospace system^14,15^. Compared to many other 3D structures, Sm offers higher surface basicity, more space and pores with a good surface morphology, faster oxygen ion mobility, the ability to trigger more redox processes, and a high specific surface area which offer extended cycle life, high specific capacitance, long-term stability, and high energy density capacity of Sm-based composites. Therefore, R. Parihar *et. al.* utilized strontium substituted SmNiO_3_ as a novel electrode materials for alkaline water electrolysis^16^. SmNiO_3_/SWCNT perovskite composite have been prepared by M. Isacfranklin *et. al.* and observed superior electrochemical properties for next generation electrodes in a hybrid supercapacitor^10^. J. Chen *et. al.* revealed the interesting effect of lattice distortion in the hydrogen-incorporated metal-insulator transition of SmNiO_3_^17^. Recently, the thermochromic characteristics of SmNiO_3_ thin films were also explored, where a reversible change in color in SmNiO_3_ was observed with the variation of temperatures. Z. F.-Gutiérrez *et. al.* deposited SmNiO_3-δ_ thin films by magnetron sputtering, crystallized by soft annealing in air, and observed thermochromicity for solar absorber^18^. Also, P.-A. Tostivint *et. al.* examined the thermochromic response in SmNiO_3_ thin films by infrared spectroscopic ellipsometry^19^. However, the electric field emission study in SmNiO_3_ has not been explored yet. Compared to the other rare-earth nickelates, SmNiO_3_ has a smaller rare-earth ionic radius, leading to stronger Ni-O-Ni bond bending, greater electron phonon coupling, and a distinctive electronic structure which strongly influences the charge transport and electric field emission response. Additionally, the SmNiO_3_ can exhibit high electrical conductivity, low work function, and sharp nanostructures with high aspect ratios, which can assist efficient electric field emission response^20,21^.

The work function (Φ), which is the smallest amount of energy required for an electron to escape into a vacuum, traps the electrons inside a conductor or semiconductor at its surface. In an electric field emission process, the potential barrier becomes thin and triangular under a very strong electric field (E) (typically ≥10^6^ V/m), and it permits electrons to tunnel through it quantum mechanically instead of thermally. The SmNiO_3_ contains mixed valence states of Ni (Ni^2+^/Ni^3+^), which enables high charge carrier mobility and strong electron delocalization at high electric field above MIT, reducing the effective work function and improving the electron tunneling probability. The mixed valence conduction is ideal for stable field emission currents. Various nanostructured morphologies of SmNiO_3_ such as nanopins, nanorods, or nanowires, can create high aspect ratios, local electric field enhancement (β) at their tips, and drastically lowered the turn-on voltages. Few electric field emission studies was observed on other rare-earth nickelates with different surface morphologies. Exciting field emission response with strong localization effect in conduction mechanism was studied on perovskites nanostructured LaNiO_3_ by R. B. Kamble *et. al*^22^. The impressive electric field emission characteristics with spin-canted magnetism and bipolaron freezing electrical transition in NdNiO_3_ was observed by S. Karmakar *et al*^23^. Recently, S. Karmakar *et. al.* also prepared GdNiO_3_ microflower by hydrothermal methods and discovered its potential field emission response^24^.

Therefore, we are motivated to grow and synthesize a unique pin-like nanostructure of SmNiO_3_ with high aspect ratios (ratios between width and height) by the hydrothermal method and investigate its structural, surface morphological, and electric field emission response with the correlation of density functional theory (DFT) study. The X-ray diffraction (XRD), Raman, X-ray photoelectron spectroscopy (XPS), and high-resolution electron microscopy techniques were utilized to confirm the phase purity, lattice vibration, chemical & oxidation states, and microstructural features of SmNiO_3_ nanopins. The electric field emission response was characterized by current density (J) vs. electric field (E) curves, Fowler-Nordheim (F-N) plot, and field emission current stability test using a planar diode configuration set-up and applying a very strong electric field. The local work function (φ) is an important parameter to determine the field enhancement factor (β), which was calculated by a DFT study using the output structural and lattice parameters obtained from a Rietveld refinement study.

## Experimental details

### Synthesis procedure

**Fig. 1:**
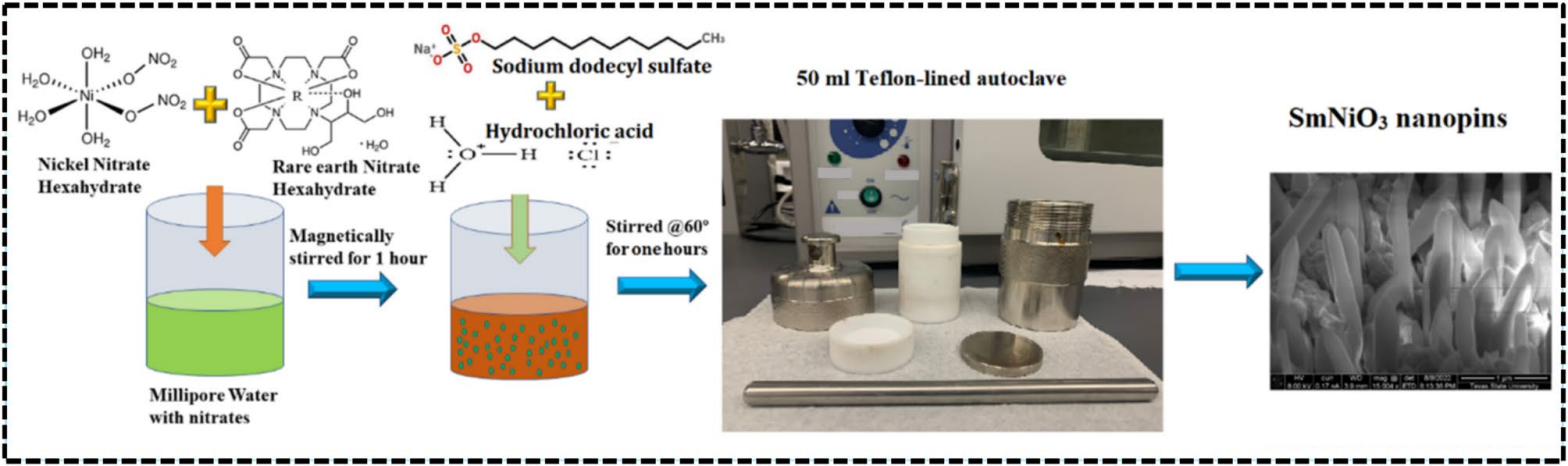
The schematic illustration of hydrothermal synthesis of 3D SmNiO_3_ nanopins.

For the synthesis of SmNiO_3_ nanopins, stoichiometric amounts of samarium nitrate hexahydrate [Sm(NO_3_)_3_·6H_2_O] and nickel (II) nitrate hexahydrate [Ni(NO₃)₂·6H₂O] were taken in 40 mL of Millipore water and mixed properly by magnetically stirring the complex in a hot oven at a temperature of 60 °C. After 1 hour of mixing, a minimum amount of hydrochloric acid (HCl) and sodium dodecyl sulfate (NaC_12_H_25_SO_4_) was added very carefully, which acts as a pH-controlling agent, oxidation-state stabilizer, and soft-template that can direct the shape and size of the nanopins of SmNiO_3_. The resultant mixture was stirred magnetically again for 1 hour, maintained a pH value of ~7.0–7.5, and transferred to a 50 ml teflon-lined stainless-steel autoclave as shown in Figure 1. The autoclave was kept in a hydrothermal microwave vacuum reactor with self-generated autoclave pressure~ 12–15 bar for 24 hours at a temperature of 120 °C. The resulting solution was collected and cleaned with distilled water (DI), ethanol, and methanol (to wash out residues), and again centrifuged to collect the precipitates. The precipitates were taken in a petridis, dried at 100 °C overnight, and the dried powder was calcined at 850 °C for 6 h to obtain the unique orthorhombic SmNiO_3_ phase. The schematic process with a real image for the synthesis of SmNiO_3_ nanopins is portrayed in Figure 1. The stoichiometry of the synthesis process was calculated following the equation, and the initial amounts taken (for 3 gm. SmNiO_3_) were mentioned in Table 1,


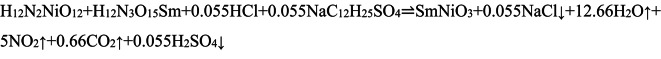


**Table 1:** The initial amounts of reactants used for the synthesis of SmNiO_3_ nanopins.

**Reactants**	**Initial amount Required**	
	**Mole**	**Gram**
H_12_N_2_NiO_12_	0.01167	3.39354
H_12_N_3_O_15_Sm	0.01167	5.18689
HCl	0.00065	0.0237
NaC_12_H_25_SO_4_	0.00065	0.18745

### Characterization techniques

The XRD data were collected by Rigaku SmartLab X-ray Diffractometer (XRD) system using a Cu-Kα source (wavelength 1.54 Å) in the range of 2θ between 10°-90°, scan rates 2°/min, and step size 0.001°, respectively. The Rietveld refinement of XRD was performed by Match and FullProf Suite software, and the output crystallographic information file (CIF) data was used for DFT calculation, maximum entropy method (MEM) pattern, and unit cell-crystal structure. The Raman spectra were obtained by Horiba LabRAM HR Evolution in the range of wavenumber 250-550 cm^-1^ using a 532 nm excitation laser source with high spectral resolution (<0.5 cm^-1^). To observe micro- and nano-structural morphology of SmNiO_3_ nanopins, the scanning electron microscopy (SEM) and transmission electron microscopy (TEM) images were acquired by Helios NanoLab 400 DualBeam FEI-SEM with focused ion beam (FIB) and resolution 1.7 nm, and FEI Talos™ f200i S/TEM is a 200 kV field emission (scanning) transmission electron microscope with magnification range 25X–1.05MX. ​An XPS-SPECS system was used to obtain the XPS data and surface information with elemental oxidation states (Sm, Ni, and O) using a monochromatic Al-Kα X-ray excitation source. We investigated the field electron emission behavior of SmNiO_3_ nanopins emitter in an ultrahigh-vacuum chamber (pressure of ~1×10^−7^ mbar) using a planar diode configuration setup. A circular indium-titanium oxide (ITO) screen with a phosphor coating (about 50 mm in diameter) served as the anode (collector), and tiny amount of synthetic SmNiO_3_ powder was applied on a tiny piece of 4 mm square conductive carbon tape to act as the cathode (emitter). The electrode spacing was carefully adjusted to ~1 mm using a linear motion motor. A 14S multimeter was utilized to measure the field-emission current that resulted from applying a potential difference between the electrodes using a dual-polarity DC power source. For the stability test, a data logger (multi S1232 Adapter, sampling interval of 10 s) was used to record the measured field emission current values over a long period of time. Electric field emission pictures created on the phosphor screen were captured by a digital camera (PowerShot SX 170 IS).

### Computational details

The Density Functional Theory (DFT) simulations for the SmNiO_3_ system under consideration were executed utilizing the Vienna Ab-initio Simulation Package (VASP) code^25,26^. Generalized Gradient Approximation (GGA and GGA+U)^27^ method coupled with a projector augmented wave (PAW) pseudopotential^28^ was used to define the exchange correlation functional, and the expansion of plane wave basis set was carried out with an energy cut off of 500 eV. The structural optimization was considered complete when the energy and Hellmann-Feynman forces reached the corresponding convergence criteria of 0.01 eV/Å and 10^-5^ eV. A K-point grid of 7×7×5 and 7×5×1 based on the Monkhorst pack method was used for bulk and surface relaxation, respectively. Moreover, a vacuum spacing of 25 Å was given in the z-direction in order to avoid the periodic image interactions.

## Experimental results & discussion

**Fig. 2:**
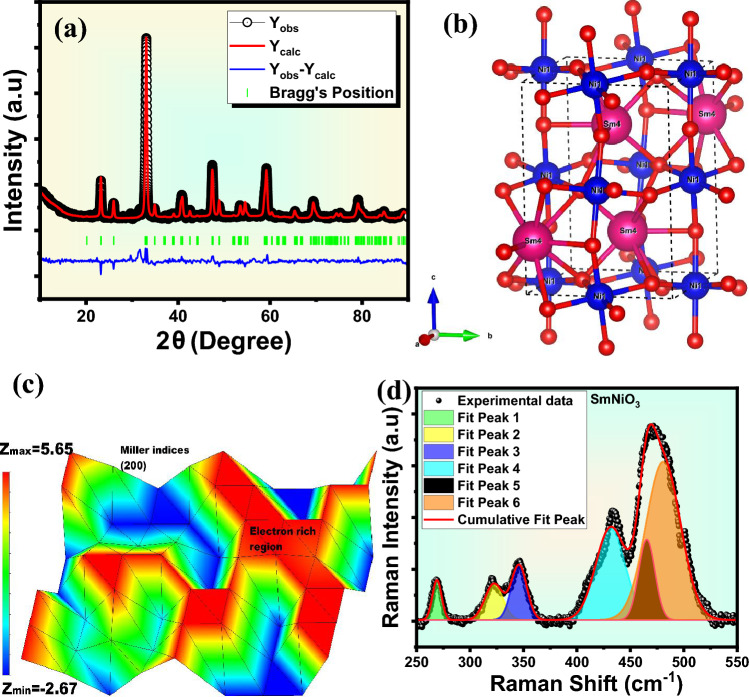
(**a**) Rietveld refinement of the X-ray diffraction (XRD) pattern, (**b**) unit cell crystal structure, (**c**) maximum entropy method (MEM) pattern along the most intense 200 peak, (**d**) room-temperature Raman spectra of SmNiO_3_ nanostructures.

The XRD spectrum is essential for confirming the phase formation, crystallinity, and crystal structure of SmNiO_3_ nanopins, and it is shown in Figure 2a with refinement. The XRD pattern exhibits all sharp, well-defined peaks indexed with a standard JCPDS card no 51-0391, corresponding to the orthorhombic phase (space group *pnma* 62) of SmNiO_3_^29–31^. Additionally, very few minor impurity peaks are detected, which correspond to unreacted residual secondary phases of Sm_2_O_3_ or NiO (collectively contribute less than 3% of the total phase fraction, while the SmNiO_3_ phase dominated >97%), oxygen vacancy/defects, or may be local structural distortions during sample preparation. The phase contributions are obtained after refinement considering all three phases and shown in the supplementary Figure S1. Under high electric fields, these secondary phases can introduce new conduction channels/pathways, heterojunctions, and localized defect states that promote electron tunneling during emission. The fitting parameters after the refinement of XRD were obtained *R*_*wp*_=14.52, *R*_*p*_ = 11.19, and goodness of fit = 1.68, and indicate a very good fitting. The refinement of the XRD pattern also divulges the refined lattice parameters of SmNiO_3_ as $a = 5.318{\mathrm{\AA}},b = 5.432{\mathrm{\AA}},c = 7.578{\mathrm{\AA}},$ , and $\alpha =\beta =\gamma =90^\circ$, volume=218.9 ${\mathrm{\AA}}$ ^3^.^32^. The structural parameters, atomic positions with Wyckoff coordinates, are also shown in Table 2. The unit-cell crystal structure of SmNiO_3_ is constructed in VESTA software using the output CIF file after Rietveld refinement and illustrated in Figure 2b. The Ni^3^⁺ ions occupy in octahedral B-sites, whereas Sm^3^⁺ ions inhabit 12-fold coordinated A-sites in the crystal structure. All Ni atoms are positioned in the center of the octahedron and O atoms in the corners of the octahedron in the 3D network of corner-sharing NiO_6_ octahedra. The overall charge is balanced by Sm atoms, which are located in the space between octahedra, and the octahedral tilt and rotate as a result of the tiny ionic radius of Sm^3^⁺, which lowers the overall symmetry from cubic to orthorhombic^33^. The bandwidth and electron-lattice coupling are greatly influenced by the Ni–O–Ni bond angle (~156.5°) and octahedral distortions, which in turn control the electronic properties and MIT in SmNiO₃^34^. Compared to traditional Fourier transform approaches, the Maximum Entropy Method (MEM) pattern is one of the more accurate and less biased computational tools that is mainly utilized in electron diffraction and XRD research to obtain electron density distributions from diffraction data. The MEM pattern gives a nice visualization of electron density distribution, which is very important for electric field emission study, and it is depicted in Figure 2c for (200) planes. The MEM is a mathematical technique that eliminates needless assumptions about the unknown structure while reconstructing the most likely electron density map that matches the experimental diffraction data (structure factors). This entropy is the information entropy, not the thermodynamic entropy, and it can be defined as^35–37^,

**Table 2:** The refined structural parameters and atomic positions of SmNiO_3_ nanopins after Rietveld refinement.

**Atom**	**Wyckoff position**	**x**	**y**	**z**	**Coordination**	**Occupancy**	**U**_**iso**_
Sm	4c	0.9884	0.0514	0.25	12-fold (Sm-O)	1	0.009
Ni	4b	0.50	0	0	6-fold (NiO_6_ octahedron)	1	0.007
O1	4c	0.0901	0.4813	0.25	Apical oxygen	1	0.012
O2	8d	0.7119	0.289	0.0426	Equatorial oxygen	1	0.014

1 $$S=-\sum {\rho }_{i}\mathrm{ln}(\frac{{\rho }_{i}}{{\tau }_{i}})$$

where ${\rho }_{i}$ is the electron density at the point *i* and ${\tau }_{i}$ is the prior (uniform) density. A high electron-rich region was observed ~65% along 220 planes of SmNiO_3,_ which assists very good electric field emission response. The room-temperature Raman spectra of SmNiO_3_ are represented in Figure 2d with different deconvoluted peaks between 250-550 cm^-1^. The broad peaks between 250 to 350 cm^-1^ are Ni-O-Ni octahedral bending modes, which are very sensitive to minor tilt/distortions. The combinations of Ni-O and Sm-O stretching/bending soft modes are visible in the range between 370 to 450 cm^-1^, and they have a significant softening effect near MIT. The broader and high-intense peaks in the higher frequency region in the range between 450-550 cm^-1^ correspond to the stronger Ni-O stretching modes in the NiO_6_ octahedra. All signature Raman peaks of SmNiO_3_ correspond to previously published work^38,39^. The coordination number of Ni and Sm was found to be 6 and 8 in the distorted NiO_6_ octahedra and SmO_x_ polyhedral cluster, respectively.

**Fig. 3:**
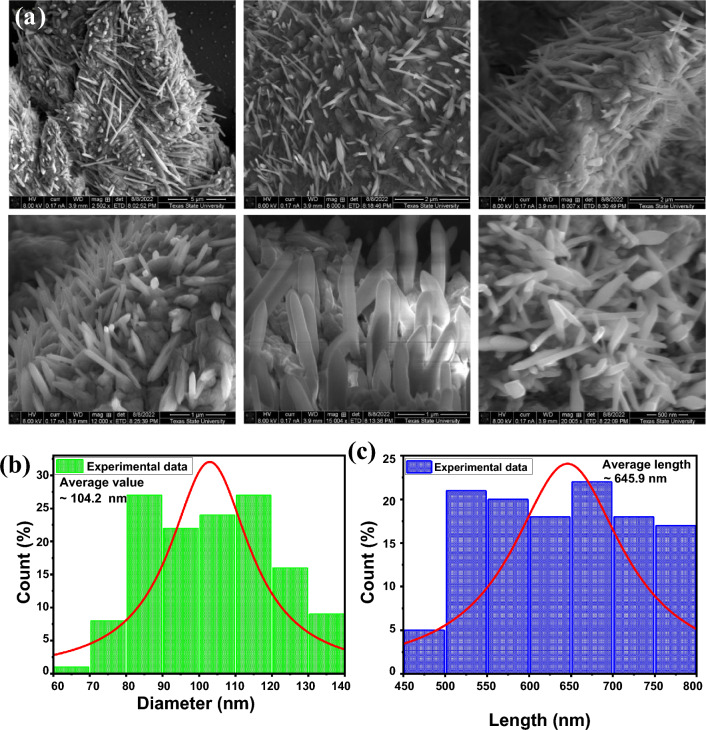
(**a**) High-resolution scanning electron microscopy (SEM) images of SmNiO_3_ nanopins at different magnification scales of 5 µm, 2 µm, 1 µm, and 500 nm. Histogram of (**b**) diameter, and (**c**) length of SmNiO_3_ nanopins over the observed area.

The high-resolution SEM micrographs of SmNiO_3_ in Figure 3a in different magnification scales (5 µm, 2 µm, 1 µm, and 500 nm) exhibit dense vertically aligned scrambled nanopins like surface morphology distributed over a dense surfactant template. From SEM micrographs, nanopins may be seen emerging from a dense lump-like formation, which is created at the first nucleation step, when the high supersaturation and high surface energy cause primary nanoparticles to clump together quickly and act as a seed platform. Most of the nanopins have a tapered morphology (sharp at the tip and thicker at the base) with a length of ~500-800 nm and a base diameter of 80-120 nm. The average value of the diameter and length was approximated to be ~104.2±0.5 nm and ~ 645.9 ±1.9 nm over the observed population and both histogram plots are shown in Figure 3b &c, respectively. Depending on the synthesis conditions and calcination temperature, they can be viewed as nanorods with tapered or pointed ends that are frequently randomly orientated or vertically aligned. The high-temperature calcination effect develops the pure phase formation of SmNiO_3_, but degrades the vertical alignment of nanopins and makes it scrambled. The high aspect ratio (h/r=20) and nanoscale sharpness of SmNiO_3_ nanopins result in significant electric field augmentation, making them ideal for field emission, gas sensing, and electrical interface applications. Surfactant-assisted anisotropic growth is commonly used to direct formation, and the resulting pin-like morphology has a high surface area, roughness, and structural stability.

**Fig. 4:**
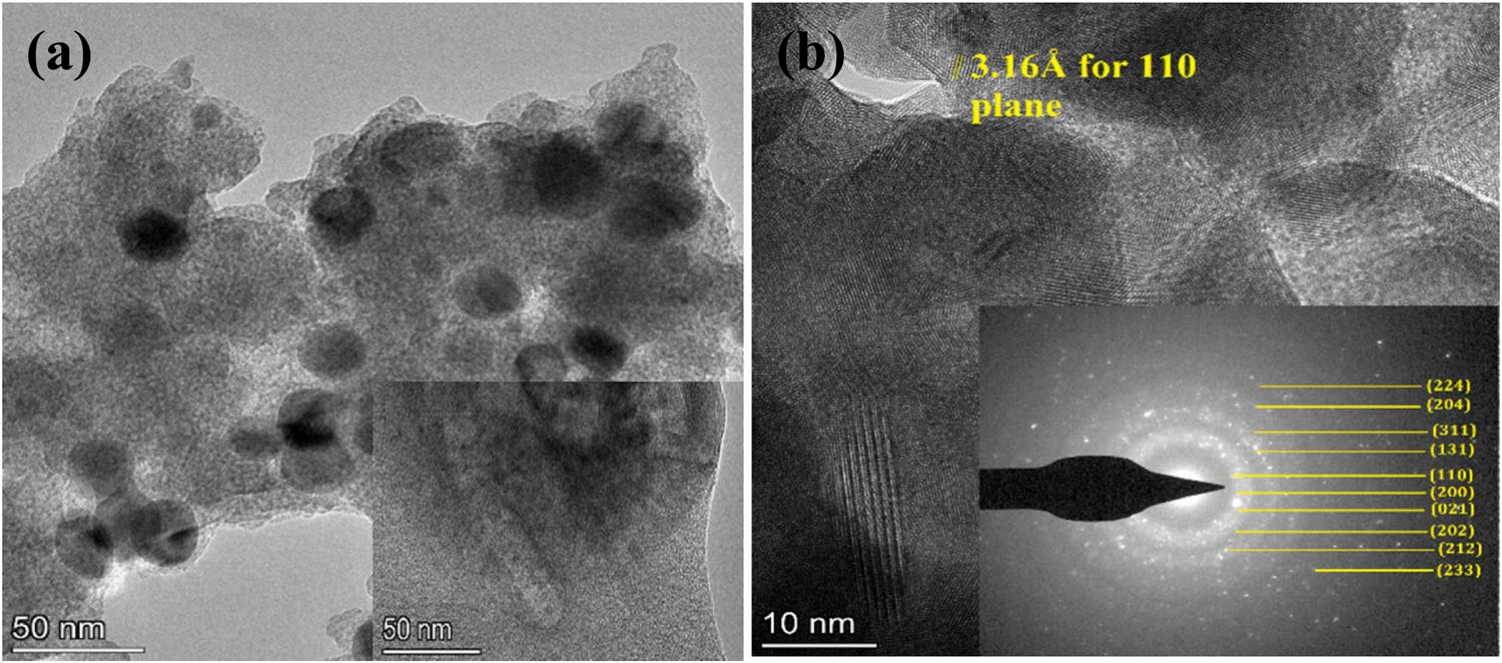
(**a**) Bright field TEM microscopy image of SmNiO_3_ nanopins, (**b**) High-resolution TEM (HRTEM) lattice fringe of SmNiO_3_ with SAED pattern.

The high-resolution TEM micrographs reveal detailed morphology, lattice fringes, and crystallinity of SmNiO_3_ nanopins, as shown in Figure 4a. The TEM image in bright field mode inside the nanopins structures reveals the circular spots with lattice fringes of different planes, confirming the polycrystalline nature of SmNiO_3_. The lattice spacing (d-value) corresponding to 110 planes is clearly visible by yellow lines in Figure 4b, which is obtained as 3.16 Å for 110 planes of orthorhombic SmNiO_3_^40,41^. The alignment of multiple planes in different orientations is also noticeable in Figure 4b, and the selected area diffraction (SAED) pattern additionally confirmed multiple circular fringes corresponding to (224)-(233) planes as shown in Figure 4b-inset.

**Fig. 5:**
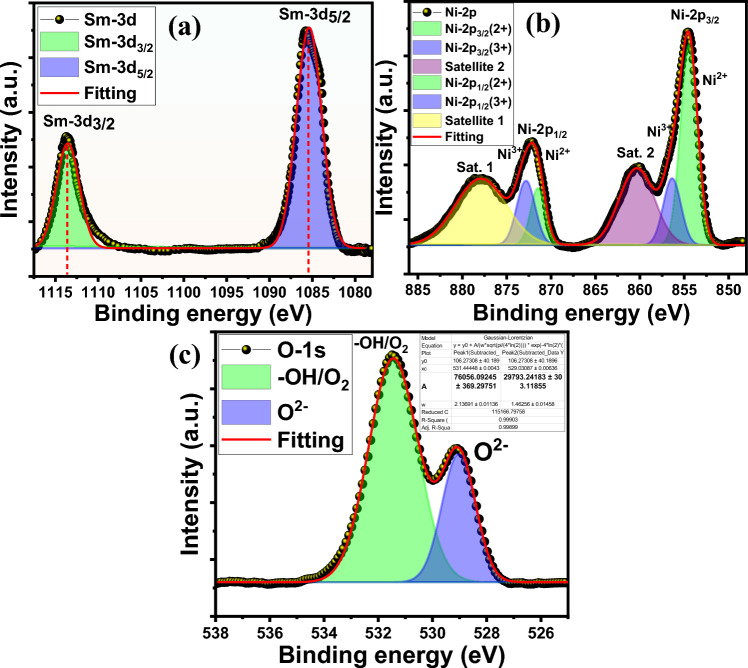
The X-ray photoelectron spectra (XPS) of (**a**) Sm-3d, (**b**) Ni-2p, and (**c**) O-1s peaks.

The XPS core-level spectra of *Sm-3d*, *Ni-2p*, and *O-1s* of SmNiO_3_ nanopins are demonstrated in Figure 5a-c, respectively, with their corresponding binding energy peaks and interpretation of chemical states. The *Sm-3d* peaks in Figure 5a exhibit two main peaks at 1086 eV and 1113 eV, which correspond to *3d*_*5/2*_ and *3d*_*3/2*_ peaks, and they are separated by ~27 eV due to spin-orbit splitting^42,43^. The *Ni-2p* spectra in Figure 5b displayed two broad and sharp peaks ~854.7 eV and ~ 872.1 eV, which were assigned to *Ni-2p*_*3/2*_ and *Ni-2p*_*1/2,*_ confirming the perovskite SmNiO_3_ stoichiometry^44,45^. Both peaks are deconvoluted into two other fitting peaks at 854.9 eV and 872.8 eV, corresponding to Ni^2+^, and 856.2 eV and 873.8 eV are associated with Ni^3+^. Two additional satellite peaks were detected ~861.5 eV and ~878 eV, emanating due to charge transfer effect or shake up processes (energy loss due to hybridization between *Ni-3d* and *O-2p*). The *O-1s* XPS spectra in Figure 5c exposed two broad and sharp peaks at ~529.3 eV and ~531.4 eV, which represent the main lattice oxygen peaks (O^2-^) bonded to Ni^3^⁺/Sm^3^⁺ and surface absorbed oxygen or hydroxyl group (sometimes indicates the oxygen vacancies), respectively^46,47^. The normalized peak-area ratio approach was used to measure the relative oxygen vacancy fraction (δ) by,

2 $$\delta =\frac{{A}_{Ov}}{{A}_{Ov}+{A}_{OL}}$$

where, ${A}_{Ov}$ and ${A}_{OL}$ are the integrated area of vacancy-related oxygen peak and lattice oxygen peaks obtained from the XPS fitting. From the fitted area of Figure 5c, ${A}_{Ov}=29793$, and ${A}_{OL}=76056$ and the value of $\delta$ was found to be ~28.1%.

**Fig. 6:**
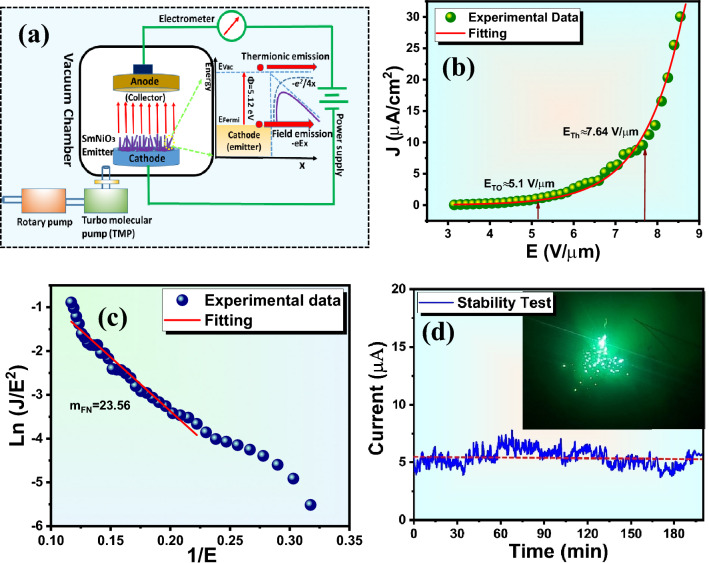
(**a**) The electric field emission set-ups with the field emission mechanism described by Fowler-Nordheim theory. (**b**) Current density (J) vs. applied electric field (E) plot, (**c**) Fowler-Nordheim (F-N) plot, (**d**) field emission current stability over a long period of 3h 20 min. Inset in Figure 5c shows the bright field emission spot collected during measurements.

The electric field emission properties of SmNiO_3_ nanopins were studied by a two-electrode diode arrangement set-up, as shown in Figure 6a. The potential energy (U(x)) of an electron just outside of a planar metal/semi-metal surface, as measured in relation to the vacuum level, is often expressed as^48,49^,

3 $$U\left(x\right)=\varphi -eEx-\frac{1}{4\pi {\varepsilon }_{0}}\frac{{e}^{2}}{4x}$$

where $\varphi$ is the work function, $-eEx$ is the linear lowering due to a uniform external electric field, and the last part is associated with the image-force potential. The potential barrier at the metal-vacuum interface narrows and becomes triangular with a strong applied electric field (~10^6^–10⁹ V/m). This enables electrons close to the Fermi level to tunnel through the barrier rather than needing sufficient energy to cross it, and the emission of electrons is purely due to an applied electric field, without thermal excitation, as shown in Figure 6a. The field emission current density (J) vs. applied electric field (E) of SmNiO_3_ nanopins is illustrated in Figure 6b, and it is fitted by the famous Fowler-Nordheim (F-N) equation. According to those laws, the current density (J) resulting from quantum mechanical tunneling of electrons over a potential barrier at a metal–vacuum or semiconductor–vacuum interface under a strong electric field and is described by^50–52^,

4 $${J}_{FN}=\lambda {a}_{FN}\frac{{E}^{2}}{\varphi }\mathrm{exp}[-\mu {b}_{FN}\frac{{\varphi }^{3/2}}{E}]$$

where,${a}_{FN}$ and ${b}_{FN}$ are the 1^st^ and 2^nd^ F-N constant having values 1.54×10^−6^ A·eV·V⁻^2^ and 6.83×10^9^ V·m⁻^1^·eV⁻^3/^^2^, respectively. The coefficients λ and µ are the generalized correction factors and both values are 1 for the ideal F-N type equation. The maximum field emission current density was observed ~ 30 µA/cm^2^ at the applied electric field ~8.4 V/µm. The turn-on (E_turn-on_) and threshold field (E_threshold_) are found to be ~ 5.1 V/µm and ~ 7.64 V/µm @10 µA/cm^2^ for drawing current density 1 µA/cm^2^ and 10 µA/cm^2^, respectively. The applied electric field is different from the local electric field at an emission site because the emitter surface becomes a little rough when SmNiO_3_ nanopins are deposited as a planar cathode. At a higher electric field, the tunneling probability as well as J increases exponentially with E, and current density can be projected to µA/cm^2^ to mA/cm^2^ at the electric field above ~9 V/µm. The field enhancement factor is the ratio of the applied average electric field to the actual local electric field. The F-N plot (plot between ln J/E^2^ *vs.* 1/E) is demonstrated in Figure 6c, and the field enhancement factor (β) is determined from the slope (m_FN_) of the F-N plot using the following formula^53,54^,

5 $$\beta =\frac{(-6.8\times 1{0}^{3}){\varphi }^{3/2}}{{m}_{FN}}$$

where *φ* is the local work function, and it is calculated to be ~5.13 eV from the DFT study, which is discussed in the next section. The $\beta$ value was calculated to be ~3343 by the eq^n^ (5) using a slope value of 23.56. The deviation from linearity in the F-N plot in the low electric field region was due to the semiconducting nature, change in local work function, adsorption/desorption of gas molecules or oxidation, variation of field enhancement and barrier shape, resistive heating, and multiple emission sites^55,56^,

A steady field emission current is necessary for the majority of applications using field emitters because variations in the emission current can result in noise, device deterioration, and inconsistent functioning, stability is crucial. The excellent field emission current stability is observed at a preset value of 5 µA, recorded over a long period of 3 h 20 min with a sampling interval of 10s, and it is shown in Figure 6d. Tang’s equation is frequently used in field emission (FE) current stability analysis to quantitatively characterize current fluctuations and distinguish intrinsic emission stability from random noise. A normalized fluctuation parameter is proposed by Tang’s *et.al.* to evaluate the FE current stability^57,58^,

6 $$\delta =\frac{1}{\overline{I} }\sqrt{\frac{\sum_{i=1}^{n}{\left({I}_{i}-\overline{I }\right)}^{2}}{N}}$$

where ${I}_{i}$ and $\overline{I }$ are the instantaneous and average emission current, N is the total number of data recorded, and $\delta$ is the relative current fluctuation, respectively. The current fluctuations were estimated ~±13.5% of the average value, and spike-like current fluctuations are seen in the stability test. The random and abrupt current jumps or spikes originated due to adsorption and desorption of residual gas molecules (O₂, H₂O, CO, etc.) on the emitter surface, changing the local work function (φ) and surface dipoles. Additionally, decomposition, ion bombardment, or Joule heating is also responsible for fluctuations and degradation in field emission stability. At low electric fields, emission behavior is influenced by semiconducting characteristics of SmNiO_3,_ such as limited free carrier concentration and trap-assisted transport, leading to single-site emission and non-linear Fowler-Nordheim (F-N) behavior. As electric fields increase, strong band bending transitions to tunneling-dominated metallic-like emission, activating multiple sites and resulting in a linear F-N region. Also, space-charge effects at low fields reduce the effective local field, especially when they become significant in semiconducting oxides and nanostructured emitters^59^. The inset of Figure 6d displays several bright emission sites from the field emission pattern, which correspond to the atomically sharp edges of SmNiO_3_ sharp edges. Table 3 represents the comparison of field emission parameters of SmNiO_3_ nanopins with other rare-earth nickelate or similar compounds.

**Table 3:** The field emission response of SmNiO_3_ nanopins with other ANiO_3_-based or similar compounds.

**Materials**	**Synthesis protocol**	**Turn-on field**	**Field enhancement factor (β)**	**Reference**
LaNiO_3_ on Ga_0.01_Zn_0.99_O (GZO) films/Si substrate	Hydrothermal method+ RF magnetron sputtering	29.2 V/μm @ (0.1 mA/cm^2^)	128	^60^
LaNiO_3_−ZnO on Ga_0.01_Zn_0.99_O (GZO) films/Si substrate	Hydrothermal method+RF magnetron sputtering	8.6 V/μm @ (0.1 mA/cm^2^)	673	^60^
La_2_NiO_4_	Sol-gel method	5.78@(1 μA/cm^2^)	1772	^61^
LaNiO_3_	Sol-gel method	16.91 V/μm @ (0.1 mA/cm^2^)	249.3	^62^
NdNiO_3_	Sol-gel method	10.5 V/μm @ (1 μA/cm^2^)	1230	^63^
GdNiO_3_	Hydrothermal method	4.6 V/μm@ (1 μA/cm^2^) or 7.12 V/μm@ (0.1 mA/cm^2^)	1753	^64^
SmNiO_3_	Hydrothermal method	5.1 V/µm@ (1 μA/cm^2^)	3343	[present study]

## Theoretical results & discussion

**Fig. 7:**
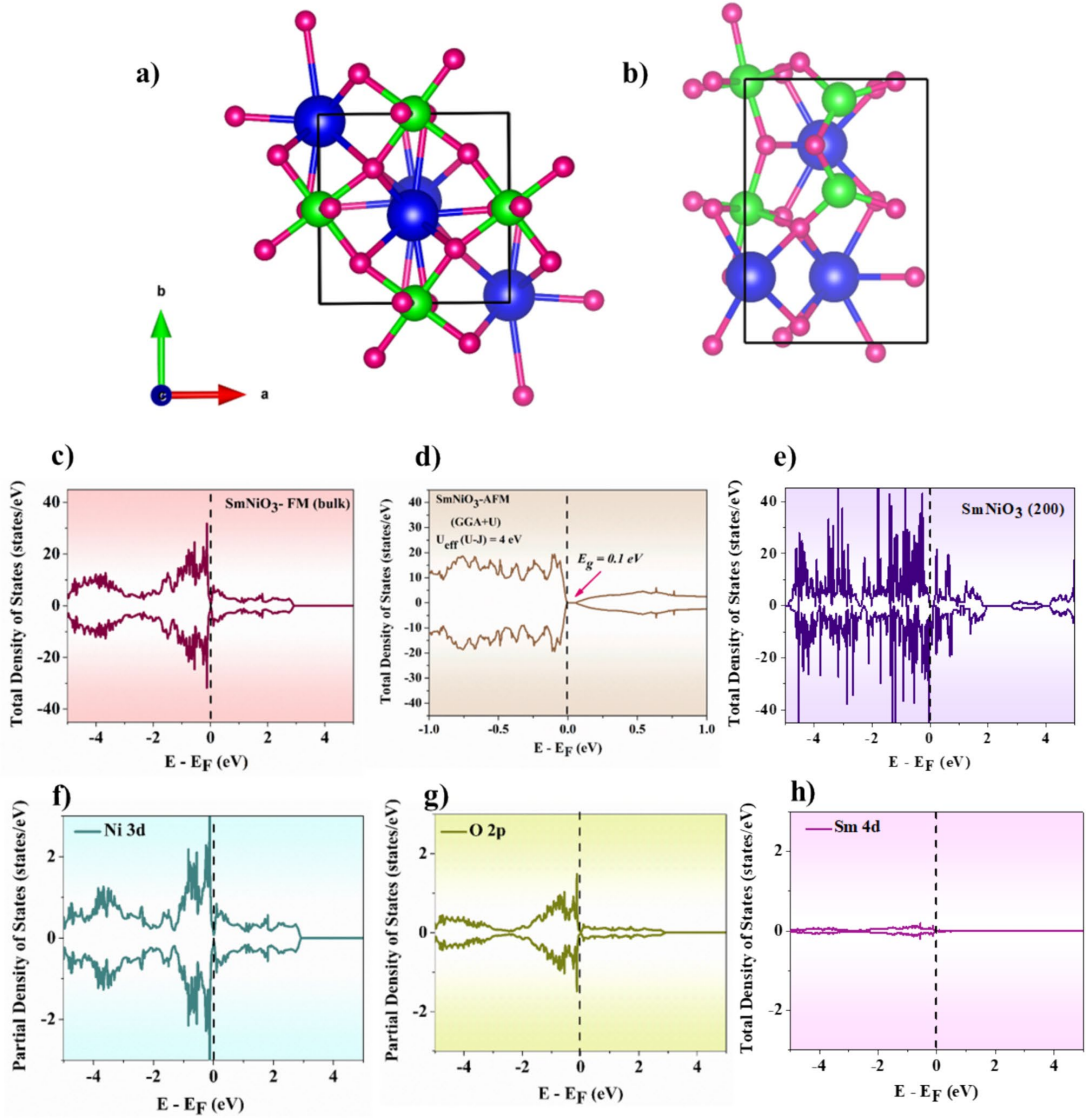
Optimized structure of (**a**) SmNiO_3_ bulk and (**b**) SmNiO_3_ (200) surface, where the blue, green, and pink colored spheres represent Sm, Ni, and O atoms, respectively. Total density of states plots for the (**c**) SmNiO_3_-FM bulk, (**d**) SmNiO_3_-AFM bulk with GGA+U correction and (**e**) (200) surface of SmNiO_3._ The dotted line drawn at 0 on the x-axis denotes the Fermi level. Partial density of states plots for (f) Ni 3d, (**g**) O 2p, and (**h**) Sm 4d orbitals of bulk SmNiO_3_.

The DFT study assists in comprehending the electronic structure, Ni-O hybridization, work function (φ), and metal-insulator transition (MIT) behavior in the rare-earth SmNiO_3_. The lattice parameter of the DFT-optimized orthorhombic bulk structure of SmNiO_3_ is a = 5.226 Å, b = 5.294 Å, c = 7.473 Å, and α = β = γ = 90°. These values match the published values of a = 5.328 Å, b = 5.437Å, and c = 7.567 Å^65^. We re-optimized the structure after cleaving the (200) surface from the bulk, which showed a strong peak in the XRD pattern. The optimized bulk and (200) surface structures of SmNiO_3_ are shown in Figure 7a&b, respectively. Further, the electronic properties of both the bulk and surface were studied by DOS calculations using the GGA exchange correlation functional. Figure 7c,d&e illustrates the total density of states (TDOS) plot of bulk SmNiO_3_-FM configuration, bulk SmNiO_3_-AFM configuration with GGA+U correction, and 200 plane of SmNiO_3,_ and it is evident that the material shows metallic nature in the bulk orthorhombic crystal structure (FM configuration), in accordance with reported literature^66^, and the (200) surface has a semi-metallic character. Additionally, calculation of DOS for SmNiO_3_-AFM configuration including GGA+U correction (U_eff_=4 eV) for *Ni 3d* orbitals reveals a band gap of 0.1 eV, matching with reported value of 0.17 eV^67^. Furthermore, the projected density of states (PDOS) plot (Figure 7f&g) of *Ni-3d* and *O-2p* in bulk SmNiO_3_ reveals that *Ni-3d* orbitals contribute more to the Fermi level. Near the Fermi level (E_F_), the substantial hybridization between *Ni-3d* and *O-2p* orbitals dominates the electronic structure of bulk SmNiO_3_. The PDOS plot of *Sm-4d* in Figure 7h lies much below the Fermi level (E_F_) and behaves as a semi-core state. The *Sm-4d* does not directly impact the conduction process, but it does have an impact on the overall charge balance and band alignment.

**Fig. 8:**
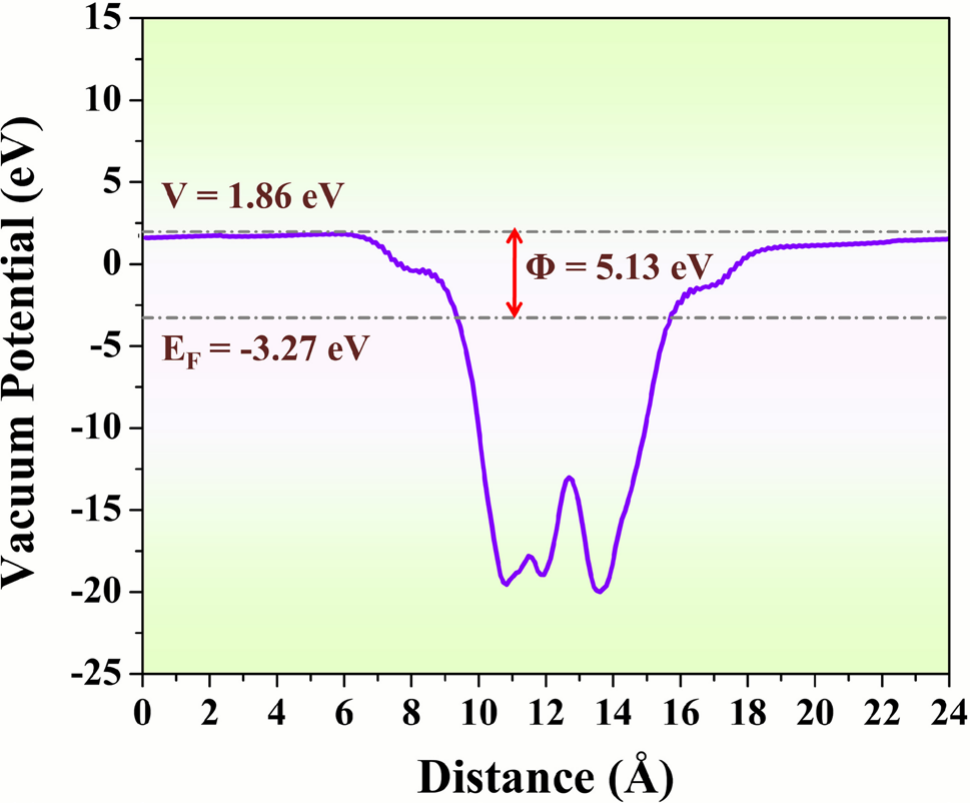
The vacuum potential (eV) plot with atomic coordinate distance (Å) for a work function of the SmNiO_3_ nanopins.

Moreover, the work function is very important for our work, and it is calculated for the (200) surface of SmNiO_3_, shown in Figure 8_._ The minimum energy required to remove an electron from the Fermi level to the surface (vacuum) of a solid material is known as the work function ${(}\varphi {)}$. The following formula can be used to calculate its value, which is extremely sensitive to the environment,

7 $$\varphi = (E_{Vac} - E_{F} )$$

where *E*_*Vac*_ and *E*_*F*_ denote the vacuum level and Fermi level, respectively. The calculated work function for the (200) surface of SmNiO_3_ is ~5.13 eV, matching the literature value of 4.93 ± 0.02 eV.^68^ The E_F_ appears negative value (-3.27 eV), which is completely normal depending on how the energy reference is chosen. DFT codes arbitrarily set the energy zero relative to the average electrostatic potential or the bottom of the valence band, and make E_F_ lie below the chosen zero potential reference.

## Conclusion

In summary, oxygen-deficient SmNiO_3_ nanopins were successfully synthesized by the hydrothermal method using sodium dodecyl sulfate (NaC_12_H_25_SO_4_) as a soft template. The Rietveld refinement of XRD confirmed orthorhombically distorted single-phase perovskite SmNiO_3_ (space group *pnma* 62) with minor secondary phases and underpins the well-formed NiO_6_ octahedral and electronic bandwidth of rare-earth nickelate. The MEM pattern exposed a rich electron density distribution along 200 planes of SmNiO_3,_ which is essential for electric field emission response. The perovskite structure of SmNiO_3_ was also validated by Raman spectroscopy by identifying different phonon modes linked to Ni–O/Sm-O bending and stretching. A dense array of high-aspect-ratio nanopins (~20) and sharp apex geometry was observed by SEM images, which is responsible for excellent field emission response. The crystal spacing 3.16 Å for 110 planes and the SAED pattern of all visible *hkl* planes corresponding to orthorhombic SmNiO_3_ was corroborated by TEM analysis. The XPS core-level spectra of *Sm-3d*, *Ni-2p*, and *O-1s* demonstrated the elemental chemical states and surface chemistry that control the field emission efficiency. By generating donor-like states and raising the near-surface carrier density, these oxygen vacancies and the corresponding changed valence states of Ni efficiently lowered the local barrier (and work function) for tunneling. The J-E curves are correlated by Fowler-Nordheim type quantum mechanical tunneling, and it exhibit low turn-on field ~5.1 V/µm at a current density of 1 μA/cm^2^ and a maximum field emission current density ~ 30 µA/cm^2^ at an electric field of ~8.4 V/µm, respectively. The field enhancement factor (β) was estimated to be 3343, and good emission current stability was observed over a long period, 3 hours and 20 minutes, with minor current fluctuations. Additionally, the DFT calculations support the experimental findings by the calculation of the density of states and work function (φ) 5.13 eV. The favorable electronic structure due to *Ni 3d*–*O 2p* hybridization and vacancy-induced donor states influenced the excellent field emission response, which recommends its potential application in field emission display and vacuum microelectronics, etc.

## Supplementary Information

Supplementary Information.

## Data Availability

Data is provided within the manuscript.
