# Supplementary material for: Fertile field emission response in scrambled SmNiO3 nanopins
Source: Sci Rep. 2026 Apr 11;16:16953. doi: 10.1038/s41598-026-37508-9 (PMC13230795; doi:10.1038/s41598-026-37508-9)
Supplement: Supplementary file 1 — Supplementary Information. [file 41598_2026_37508_MOESM1_ESM.docx]

**Supplementary material**

**Fertile Field Emission Response in Scrambled SmNiO_3_ Nanopins**

Subrata Karmakar^a,b*^, G. Anil Kumar^c^, K. Kumar Angajala^d^, Chetan D. Mistari^e^, Amrutha M^f^, **Pallab Kumar Sarkar^b^**, **Saif Taqy^b^,** Brahmananda Chakraborty^g,h^, M. A. More^i^, [Ariful Haque](https://ahaque.wp.txstate.edu/dr-ariful-haque/)^b,j^

*^a^Department of Physics, Manipal University Jaipur, Jaipur, Rajasthan 303007, India*

*^b^Electrical Engineering, Ingram School of Engineering, Texas State University, San Marcos, TX 78666, USA*

*^c^Department of Physics, Sreenidhi Institute of Science and Technology, JNTU, Hyderabad-501 301, India*

*^d^Department of Chemistry, Vardhaman College of engineering, Shamshabad, Hyderabad, Telangana 501218, India*

*^e^R. H. Sapat College of Engineering, Nashik, Maharashtra 422005, India*

*^f^Department of Physics, Karpagam Academy of Higher Education (Deemed to be University), Coimbatore, India. 641021*

*^g^Homi J Bhabha National Institute, Bhabha Atomic Research Centre, Trombay, Mumbai-400085, India****.***

*^h^High Pressure & Synchrotron Radiation Physics Division, Bhabha Atomic Research Centre, Trombay, Mumbai- 400085, India.*

*^i^Department of Physics, Savitribai Phule Pune University, Pune 411 007, India*

*^j^Materials Science, Engineering & Commercialization Program, Texas State University, San Marcos, TX 78666, USA*

*Correspondence e-mail:*[*subrata.karmakar@jaipur.manipal.edu,*](mailto:subrata.karmakar@jaipur.manipal.edu,) [*skarmakarph@gmail.com*](mailto:skarmakarph@gmail.com)


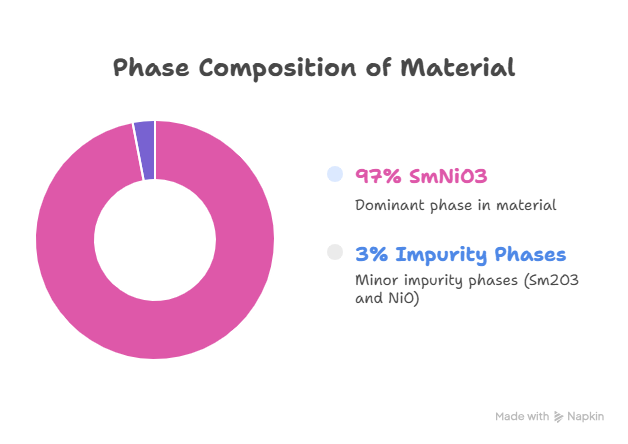


**Figure S1:** *The Rietveld refinement of the X-ray diffraction (XRD) pattern of SmNiO_3_ (97%) with another small Sm_2_O_3_, and NiO phase (3%).*
